# Topical and systemic immunoreaction triggered by intravesical chemotherapy in an N-butyl-N-(4-hydroxybutyl) nitorosamine induced bladder cancer mouse model

**DOI:** 10.1371/journal.pone.0175494

**Published:** 2017-04-13

**Authors:** Shunta Hori, Makito Miyake, Yoshihiro Tatsumi, Sayuri Onishi, Yosuke Morizawa, Yasushi Nakai, Nobumichi Tanaka, Kiyohide Fujimoto

**Affiliations:** Departments of Urology, Nara Medical University, Nara, Japan; University of South Alabama Mitchell Cancer Institute, UNITED STATES

## Abstract

Intravesical bacillus Calmette-Guerin (BCG) treatment is the most common therapy to prevent progression and recurrence of non-muscle invasive bladder cancer (NMIBC). Although the immunoreaction elicited by BCG treatment is well documented, those induced by intravesical treatment with chemotherapeutic agents are much less known. We investigated the immunological profiles caused by mitomycin C, gemcitabine, adriamycin and docetaxel in the N-butyl-N-(4-hydroxybutyl) nitrosamine (BBN)-induced orthotopic bladder cancer mouse model. Ninety mice bearing orthotopic bladder cancer induced by BBN were randomly divided into six groups and treated with chemotherapeutic agents once a week for four weeks. After last treatment, bladder and serum samples were analyzed for cell surface and immunological markers (CD4, CD8, CD56, CD204, Foxp3, and PD-L1) using immunohistochemistry staining. Serum and urine cytokine levels were evaluated by ELISA. All chemotherapeutic agents presented anti-tumor properties similar to those of BCG. These included changes in immune cells that resulted in fewer M2 macrophages and regulatory T cells around tumors. This result was compatible with those in human samples. Intravesical chemotherapy also induced systemic changes in cytokines, especially urinary interleukin (IL)-17A and granulocyte colony stimulating factor (G-CSF), as well as in the distribution of blood neutrophils, lymphocytes, and monocytes. Our findings suggest that intravesical treatment with mitomycin C and adriamycin suppresses protumoral immunity while enhancing anti-tumor immunity, possibly through the action of specific cytokines. A better understanding of the immunoreaction induced by chemotherapeutic agents can lead to improved outcomes and fewer side effects in intravesical chemotherapy against NMIBC.

## Introduction

Urothelial carcinoma of the bladder (UCB) is the fourth and eighth most common malignancy in men and women, respectively, in the United States. It is estimated that, of the 74,690 patients who were diagnosed with UCB in 2014, 15,580 died of the disease [1]. Approximately 70% of UCB cases are diagnosed as non-muscle invasive bladder cancer (NMIBC), including stages Ta and T1 [2, 3]. NMIBC is treated by transurethral resection of the bladder tumor (TURBT), followed by the administration of adjuvant intravesical treatment with bacillus Calmette-Guerin (BCG) or chemotherapeutic agents, such as adriamycin (ADM), mitomycin C (MMC), gemcitabine (GEM), or docetaxel (DTX) [4, 5]. These intravesical treatments can decrease recurrence rates and prolong the progression-free interval [6, 7]. However, NMIBC has a significant potential to progress to muscle invasive bladder cancer (MIBC) after initial treatment, requiring invasive procedures such as radical cystectomy. T1 high-grade bladder cancer progresses to MIBC at a rate of 25% to 50% [8, 9] and Ta high-grade bladder cancer presents a recurrence rate of 50% to 60% in spite of adjuvant intravesical treatment [10]. The main problem is that NMIBC can be lethal at varying degrees of progression [8, 9, 11] and, in the case of recurrence, patients need to undergo repeated rounds of TURBT. The aim of NMIBC clinical management is to prevent cancer progression and recurrence.

Intravesical treatment with BCG represents the most effective and common form of adjuvant therapy for high risk NMIBC [12]. BCG binds to the urothelial lining and then elicits a nonspecific immune response within the bladder wall, involving the activation of multiple types of immune cells and cytokines. BCG is processed by normal and malignant cells to trigger a complex pro-inflammatory response characterized by release of interleukin (IL)-1, IL-6, IL-8, tumor necrosis factor (TNF)- α, and granulocyte macrophage colony stimulating factor (GM-CSF) [13, 14]. This response is T lymphocyte-dependent and is mediated by both T helper 1 (Th1) and 2 (Th2) cytokines. These, in turn, stimulate lymphokine-activated killer cells, NK cells, macrophages, neutrophils, as well as TNF-related apoptosis-inducing ligand (TRAIL) and fatty-acid synthase (Fas) ligand apoptotic pathways [15–17]. Although some reports have associated urinary Th1 cytokines with the BCG response, others have correlated high levels of Th2 cytokines with BCG failure [18–20].

Anti-NMIBC chemotherapeutic agents include alkylating compounds, topoisomerase inhibitors, and deoxycytidine analogs, among others. The association between intravesical treatment with chemotherapeutic agents and systemic and/or local immunoreactions has not been established yet. Elucidation of the underlying mechanism may lead to the discovery of novel UCB biomarkers with improved accuracy in predicting cancer progression or recurrence. Moreover, novel chemotherapeutic agents for intravesical treatment of NMIBC may benefit from fewer side effects. The aim of the present study was to evaluate immune-related cells and cytokines, which might be induced by intravesical treatment with chemotherapeutic agents.

## Material and methods

### Animals

Ninety 6-week-old C57BL/6J female mice were obtained from Oriental Bio Service (Kyoto, Japan). Animal care was in compliance with the recommendations of The Guide for Care and Use of Laboratory Animals (National Research Council) and the study was approved by the animal facility committee at Nara Medical University (ID: 11326).

### Reagents

To prepare an orthotopic bladder cancer model we used N-butyl-N-(4-hydroxybutyl) nitrosamine (BBN, B0938; Tokyo Chemical Industry, Tokyo, Japan). To treat BBN-induced bladder cancer with intravesical treatment the following chemotherapeutic agents commonly used in clinical intravesical treatment of NMIBC were prepared: BCG (Nippon Kayaku, Tokyo, Japan), MMC (Kyowa Hakko Kirin, Tokyo, Japan), ADM (Wako, Osaka, Japan), GEM (Taiho Pharmaceutical, Tokyo, Japan), and DTX (Yakult Pharmaceutical Industry, Tokyo, Japan). All agents were diluted in sterile saline solution. Sterile phosphate-buffered saline (PBS) was used as control.

### Bladder tumor model in mice and intravesical treatment

**Fig 1:**
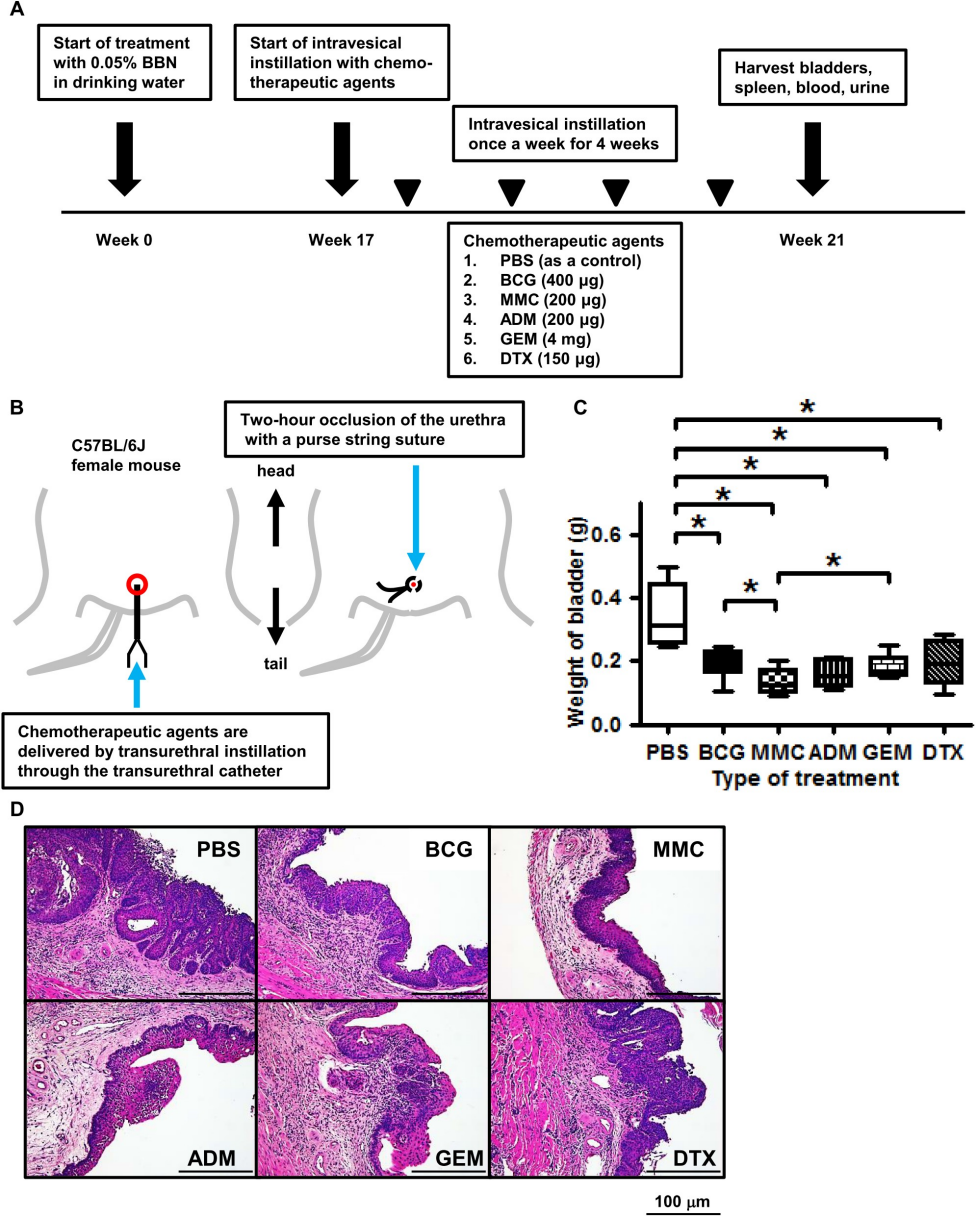
Study treatment schema and antitumor activity in BBN-induced mause bladder cancer model. **(A)** Schematic diagram illustrating the study work flow. Mice were given 0.05% BBN in drinking water, continuously for 16 weeks, and were then randomly divided into six groups (N = 15 per group). Starting with week 17, mice were treated once a week for 4 weeks with PBS, BCG, MMC, ADM, GEM, and DTX. One week after the last treatment, mice were euthanized and bladder, spleen, whole blood by cardiac puncture, and urine were harvested. **(B)** Illustration showing the procedure of intravesical instillation and occlusion of the urethra with a purse string suture. Chemotherapeutic agents are delivered by transurethral instillation through a catheter allowed to dwell in the bladder for 2 h. **(C)** Intravesical treatment with chemotherapeutic agents causes significant bladder weight loss compared to control (Mann-Whitney U test; * = *P*< 0.05). **(D)** Representative images of hematoxylin-eosin-stained bladder samples from each group. All chemotherapeutic agents have anti-tumor effect similar to BCG treatment in orthotopic model.

Fig 1A shows a schematic representation of the study. After allowing mice to acclimate to our facility for one week, they received 0.05% BBN in drinking water, continuously for 16 weeks, to induce the development of NMIBC. Next, mice were allowed to resume drinking BBN-free water. We randomly divided the mice into six groups (N = 15 mice per group): control (PBS), BCG (400 μg/dose), MMC (200 μg/dose), ADM (200 μg/dose), GEM (4 mg/dose), and DTX (150 μg/dose). Intravesical treatment was initiated from week 17 and given once a week for four weeks. Fig 1B illustrates the procedure of catheterization and occlusion with a purse string suture. Briefly, all bladder instillations were performed under anesthesia with isoflurane, whereby a 24-gauge Teflon angiocatheter was placed into the bladder via the urethra. Urine was completely drained from the bladder and collected. Next, PBS, BCG, MMC, ADM, GEM, and DTX were delivered by transurethral instillation through the catheter and allowed to dwell in the bladder by occlusion of the urethra with a purse string suture. After 2 h, the suture was removed and mice were stimulated to expel bladder contents. One week after the last intravesical treatment all mice were euthanized by exsanguination under anesthesia with isoflurane and tissues (bladder, spleen, whole blood by cardiac puncture, and urine) were harvested. All of the procedures of dealing with mouse were performed under anesthesia with isoflurane and all efforts to minimize suffering of them were made such as enough sedation, reduction of treatment time, and so on.

### Immunohistochemistry (IHC) staining

All resected bladders were filled with 150 μL 10% neutral buffered formalin and the entire specimens, along with resected spleens, were placed in 10% neutral buffered formalin. Bladders and spleens in formalin were embedded in paraffin and then subjected to IHC staining for cell surface and immunological markers Cluster designation (CD)4, CD8 (both T cells), CD56 (NK cells), CD204 (M2 macrophages), Forkhead box p3 (Foxp3) (regulatory T cells, Tregs), and Programmed cell death-ligand 1 (PD-L1). Paraffin blocks were cut and placed on Superfrost Plus microslides (Thermo Fisher Scientific, Yokohama, Japan). Sections were deparaffinized and citric acid buffer (pH 6.0) antigen retrieval was carried out using an autoclave. IHC staining was performed using the Histofine ABC kit (Nichirei Biosciences, Tokyo, Japan) according to manufacturer instructions. Briefly, slides were incubated overnight at 4°C with mouse monoclonal antibodies against CD4 (clone 4B12, ready-to-use; Nichirei Biosciences), CD8 (clone C8/144B, ready-to-use; Nichirei Biosciences), CD56 (MA1-70100, 1:500 dilution; Thermo Fisher Scientific), CD204 (clone SRA-E5, 1:2000 dilution; Trans Genic, Kobe, Japan), Foxp3 (clone 236A/E7, 1:500 dilution; Abcam, Cambridge, UK), and PD-L1 (clone 130021, 1:50 dilution; R&D Systems, Minneapolis, MN, USA). The slides were counterstained with Mayer's hematoxylin, dehydrated, and sealed with a cover slide. In each case, positive cells were evaluated by two investigators (MM and YT), blinded to the information pertaining the treatment. Positive cells from each specimen were counted from a minimum of four randomly selected fields per high power field (HPF) and compared with the control by calculating the average number of cells.

### Immunohistochemical staining analysis of human bladder cancer tissue

To confirm in human bladder cancer, IHC staining was performed using paraffin-embedded bladder cancer tissues, which were obtained by TURBT from the patients with NMIBC. Total 12 patients who underwent adjuvant intravesical treatment of BCG, MMC, or ADM were selected (N = 4 patients per group). All patients were performed intravesical treatment immediately after initial TURBT between April 1996 and March 1998. They were all diagnosed as pathological T1 category. Median age of BCG, MMC, and ADM group is 72, 69, and 73, respectively. Male/female ratio of BCG, MMC, and ADM group is 1/1, 3/1, and 3/1, respectively (S1 Table). Primary tumors and recurrent tumors after intravesical treatment were immunostained with antibodies specific to CD204 (1:2000 dilution) and Foxp3 (1:200 dilution). The procedure of IHC staining was as described above. Positive cells were counted in 4 independent HPFs and the number of cells was calculated. We compared the number of M2 macrophages and Tregs in each treatment group before and after intravesical treatment. The protocol for the research project was approved by the Institutional Review Board for Clinical Studies (Medical Ethics Committee ID: NMU-1256), which waived the requirement for informed patient consent due to the retrospective nature of the analysis.

### Measurement of serum and urine inflammatory cytokines by ELISA

Serum was collected from every mouse in tubes, centrifuged at 10,000 × *g* for 15 min, and the supernatant was stored at -80°C. Urine was collected in Eppendorf tubes on ice containing a concentrated urine stabilizer solution (2 M Tris-HCl [pH 7.6], 5% BSA, 0.1% sodium azide, and a cOmplete mini protease inhibitor tablet [Roche Diagnostics, Indianapolis, IN, USA]). Urine was collected as one sample in five mice, thus three samples were harvested in each group. Urine was centrifuged and the supernatant was stored at -80°C. The profiles of 12 inflammatory cytokines in serum were determined using an ELISA array (MEM-004A; QIAGEN, Hilden, Germany) according to manufacturer specifications. Briefly, serum samples were incubated in 96-well microplates coated with anti-mouse primary antibodies against 12 inflammatory cytokines: IL-1A, IL-1B, IL-2, IL-4, IL-6, IL-10, IL-12, IL-17A, interferon (IFN)-γ, TNF-α, granulocyte colony stimulating factor (G-CSF), and GM-CSF. Samples were developed with horseradish peroxidase-conjugated secondary antibodies. After adding the substrate and stop solution, a TECAN microplate reader (San Jose, CA, USA) was used to measure absorbance at 450 nm. Voided urine samples of each mouse collected at every treatment were thawed just before use and changes in the concentrations of IL-17A (Platinum ELISA, Affymetrix eBioscience, San Diego, CA, USA) and G-CSF (RayBiotech, Norcross, GA, USA) were measured over time using a TECAN reader as above.

### Measurement of blood cell numbers

Whole blood was collected in tubes containing EDTA and was immediately shipped on ice to the Nagahama Life Science Laboratory (Shiga, Japan), where populations of neutrophils, lymphocytes, and monocytes were counted.

### Statistical analysis

Statistical analyses and figure plotting were performed using GraphPad Prism 5.0 (GraphPad Software, San Diego, CA, USA). The bladder weight and expression levels of each marker in mouse and human samples were compared between control and chemotherapeutic agents using the Mann-Whitney U test. The number of each blood cell was also compared between control and chemotherapeutic agents using the Mann-Whitney U test. The urine concentration of IL-17A and G-CSF was compared between control and chemotherapeutic agents using the Student’s *t*-test. A *P*-value < 0.05 was considered statistically significant.

## Results

### Anti-tumor activity of intravesical treatment with BCG, MMC, ADM, GEM, and DTX

Mice bearing bladder tumors were treated intravesically for four weeks with PBS, BCG, MMC, ADM, GEM, or DTX (Fig 1A). Maximum tumor size observed in this study is 20 mm in diameter in the control. Other than minor hematuria, treatments were well tolerated with no appreciable toxicity and body weight loss. One week after treatment completion, significant bladder weight loss was observed in the BCG, MMC, ADM, GEM, and DTX treatment groups compared with the control (*P*<0.01, *P*<0.01, *P*<0.01, *P*<0.01, *P*<0.01, respectively; Fig 1C, S2 Table). MMC had the greatest effect on bladder weight loss. Fig 1D shows representative hematoxylin-eosin-stained samples from each treatment group.

### Immune-related cells recruited by intravesical treatment of chemotherapeutic agents

**Fig 2:**
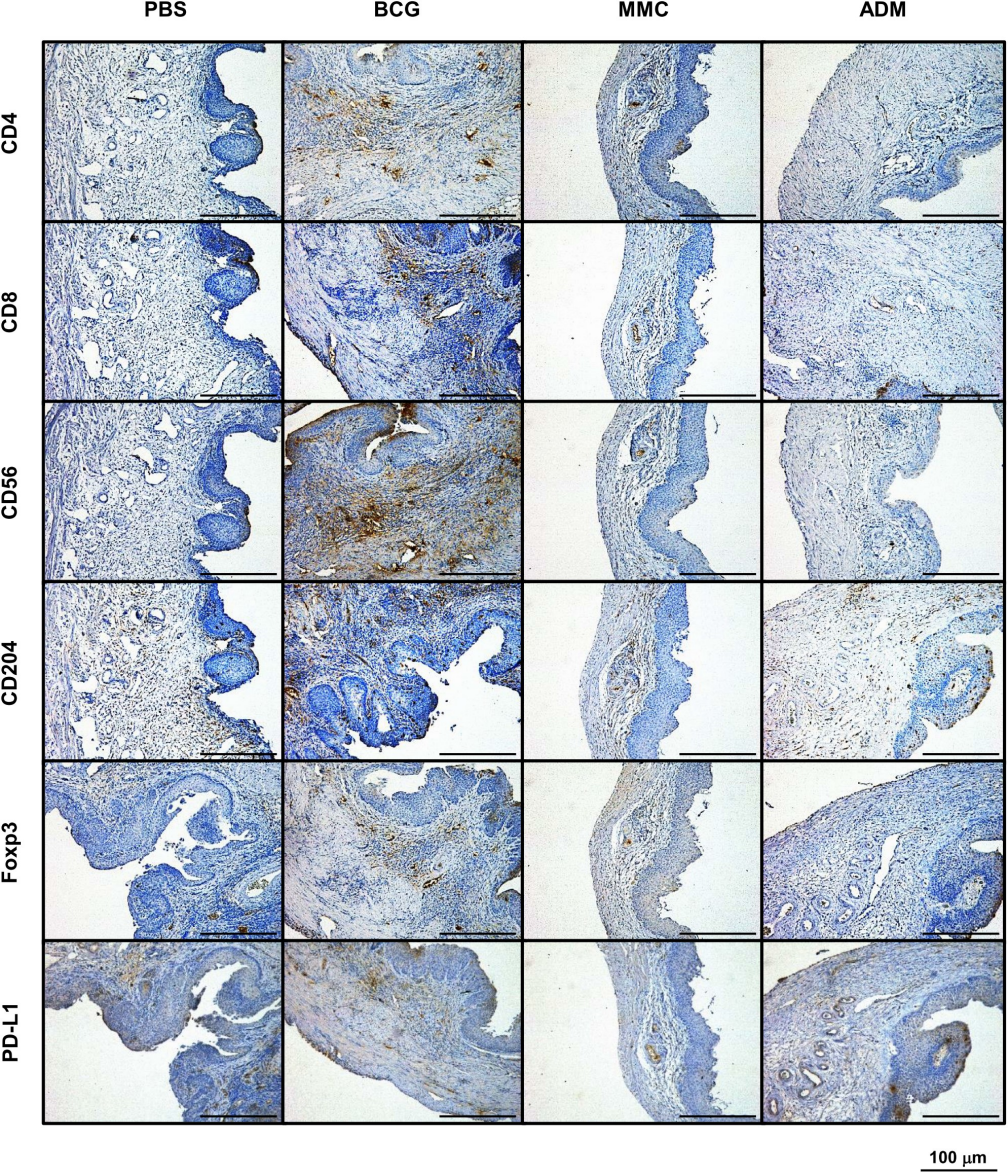
Representative images of bladder samples from each treatment group stained with six immunological markers. Tumoral infiltration of cells positive for CD4, CD8, CD56, CD204, Foxp3, and PD-L1 were noted. Expression level of each marker compared to control. CD4+ T cells, CD8+ T cells and NK cells were induced by BCG. Tregs and TAM were decreased in MMC and ADM.

To investigate the influence of various chemotherapeutic agents on immune-related cells, we performed IHC staining for six markers: CD4, CD8, CD56, CD204, Foxp3, and PD-L1. Representative images of antibody-stained resected bladders are shown in Fig 2 and results are summarized in Table 1. Treatment with GEM and DTX induced no significant change (data not shown). CD4+ and CD8+ T cells were significantly induced in the BCG group (*P*<0.01, *P*<0.01, respectively; S1A and S1B Fig, S3 Table), but not in any other group. NK cells were induced in the BCG and reduced in the MMC groups (*P*<0.01 and *P*<0.01, respectively; S1C Fig, S3 Table). M2 macrophages were significantly reduced in the MMC and ADM groups (*P*<0.01 and *P*<0.01, respectively; S1D Fig, S3 Table), as were also Tregs (*P*<0.01 and *P*<0.01, respectively; S1E Fig, S3 Table). None of the groups presented any significant change in PD-L1 positive cells (S1F Fig, S3 Table). Interestingly, MMC and ADM displayed similar immunological profiles, except for the recruitment of NK cells to the bladder wall. On the contrary, GEM and DTX had no apparent effect on immune-related cells.

**Table 1:** The summary of immunohistochemical staining analysis and ELISAaray of serum.

Treatment	Immunohistochemical staining analysis of the treated bladder						ELISA of serum
	(*vs* PBS control)						(*vs* PBS control)
	CD4	CD8	CD56	CD204	Foxp3	PD-L1	Elevated cytokines and chemokines
**BCG**	**up**	**up**	**up**	**ns**	**ns**	**ns**	**IL-1A, IL-2, IL-17A, G-CSF**
**MMC**	**ns**	**ns**	**down**	**down**	**down**	**ns**	**IL-4, IL-17A, G-CSF**
**ADM**	**ns**	**ns**	**ns**	**down**	**down**	**ns**	**IL-1A, G-CSF**
**GEM**	**ns**	**ns**	**ns**	**ns**	**ns**	**ns**	**IL-1A, IL-4**
**DTX**	**ns**	**ns**	**ns**	**ns**	**ns**	**ns**	**IL-1A, IL-10, G-CSF**

ADM = adriamycin; BCG = bacillus Calmette-Guerin; DTX = docetaxel; G-CSF = granulocyte colony stimulating factor; GEM = gemcitabine; IL = interleukin; MMC = mitomycin C; up = significantly increased compared to PBS control (P<0.05); down = significantly decreased compared to PBS control (P<0.05); ns = not significant compared to PBS control; PBS = phosphate buffered saline; Mann-Whitney U test

S2 Fig shows representative images of antibody-stained resected spleens. Here, IHC staining revealed that CD4+ T cells were induced in the BCG, MMC, and DTX groups (*P*<0.01, *P* = 0.018, and *P* = 0.021, respectively; S3A Fig, S4 Table); whereas NK cells were induced in the BCG, MMC, ADM, and GEM groups (*P*<0.01, *P*<0.01, *P*<0.01, and *P*<0.01, respectively; S3C Fig, S4 Table). No significant changes were found for other immunological markers in any of the treatment groups.

**Fig 3:**
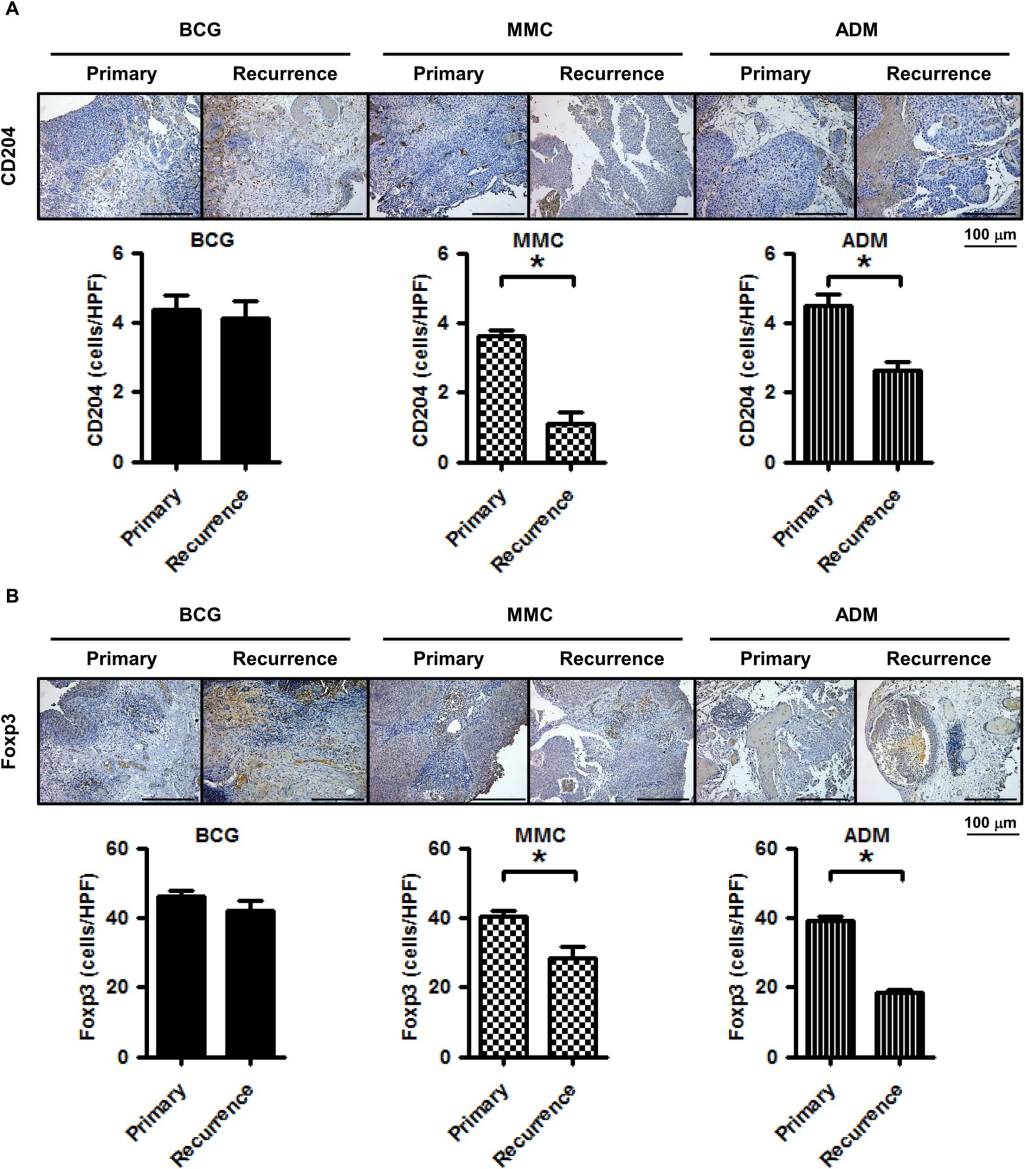
Representative images of human bladder cancer tissues from each treatment group stained with two immunological markers. Tumoral infiltration of cells positive for CD204 and Foxp3 were noted. **(A)** M2 macropages are reduced after intravesical treatment with MMC and ADM compared to primary tumors (Mann-Whitney U test; * = *P*<0.05). **(B)** Tregs are reduced after intravesical treatment with MMC and ADM compared to primary tumors (Mann Whitney U test; * = *P*<0.05).

Fig 3 shows representative images of the staining of human bladder cancer tissues. IHC analysis revealed that M2 macrophages were significantly reduced in patients treated with MMC and ADM (*P*<0.01 and *P*<0.01, respectively; Fig 3A, S5 Table) and after treatment the number of M2 macrophages were significantly reduced in these groups compared to BCG group (*P*<0.01 and *P*<0.01). Moreover, Tregs were significantly reduced in patients treated with MMC and ADM (*P*<0.01 and *P*<0.01, respectively; Fig 3B, S5 Table). These results were compatible with those in the orthotopic mice model.

### Systemic changes caused by intravesical treatment in inflammatory cytokines in serum

**Fig 4:**
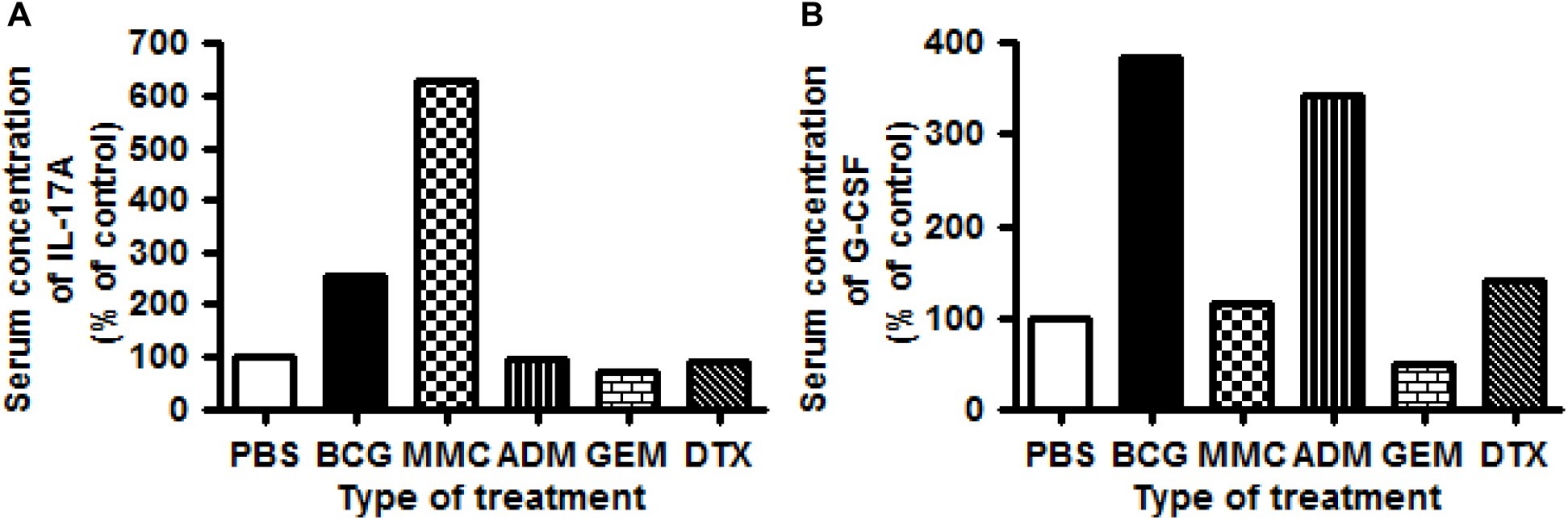
Relative concentration of serum cytokines as determined by the ELISArray Kit. (A) IL-17A is induced by BCG and MMC. (B) G-CSF is induced by BCG, MMC, ADM, and DTX.

To investigate the association between intravesical treatment and the type of induced inflammatory cytokines, we analyzed the serum obtained from the euthanized mice by ELISA. IL-1B, IL-6, IL-10, IL-12, IFN-γ, TNF-α, and GM-CSF did not present any significant change (Table 1). In contrast, IL-17A showed a 2.5-fold and 6.3-fold increase in the BCG and MMC groups, respectively, compared with the control (Fig 4A). Similarly, G-CSF displayed a 3.8-fold, 1.2-fold, 3.4-fold, and 1.4-fold increase in the BCG, MMC, ADM, and DTX groups, respectively, compared with the control (Fig 4B). Both IL-17A and G-CSF showed no increase in the GEM group.

### Changes to inflammatory cytokines in urine induced by intravesical treatment

**Fig 5:**
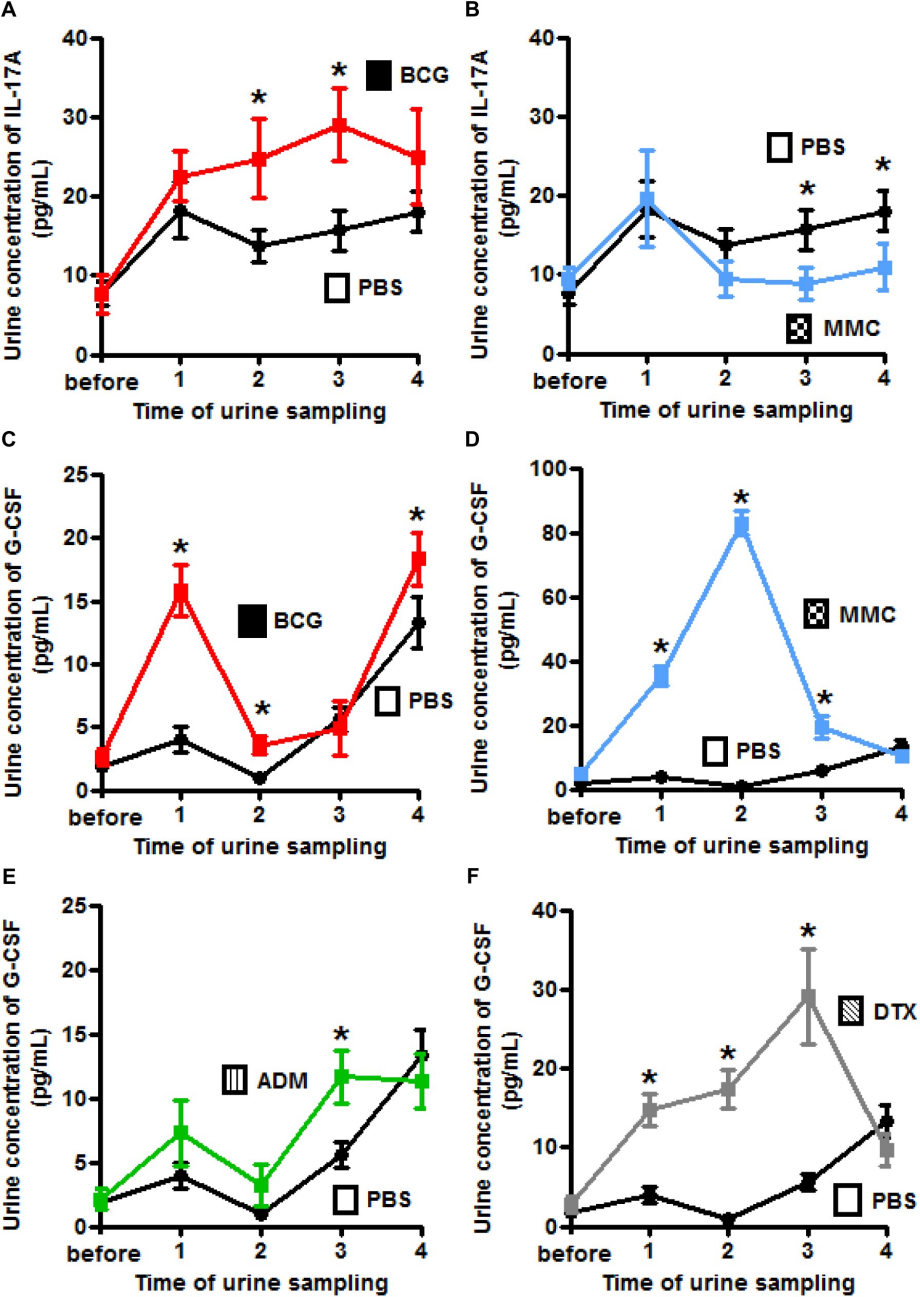
Time-course analysis of urinary IL-17A and G-CSF levels by ELISA. **(A, B)** IL-17A displays a gradual increase with BCG and decrease with MMC. **(C, D, E, F)** G-CSF shows an increase with BCG, ADM, DTX, and especially MMC (Student’s *t*-test; * = *P*<0.05).

Treatments that influenced urine concentrations of IL-17A and G-CSF were further studied by time-course ELISA analysis of urine samples (S6 and S7 Tables). Compared with the control, intravesical treatment with BCG caused a significant increase in IL-17A at the time of the second and third treatment (Fig 5A) and intravesical treatment with GEM caused a significant increase in IL-17A at the time of the first and second treatment. Instead, MMC caused a significant decrease in IL-17A at the time of the third and fourth treatment (Fig 5B). Intravesical treatment with BCG caused a significant increase in G-CSF in urine at the time of the first, second, and fourth treatment (Fig 5C). MMC achieved a comparable result at the time of the first, second and third treatment (Fig 5D). Similarly, ADM and particularly DTX led to a high G-CSF concentration at the time of the first, second, and third treatment (Fig 5E and 5F, respectively).

### Effect of intravesical treatment on the population of blood cells

**Fig 6:**
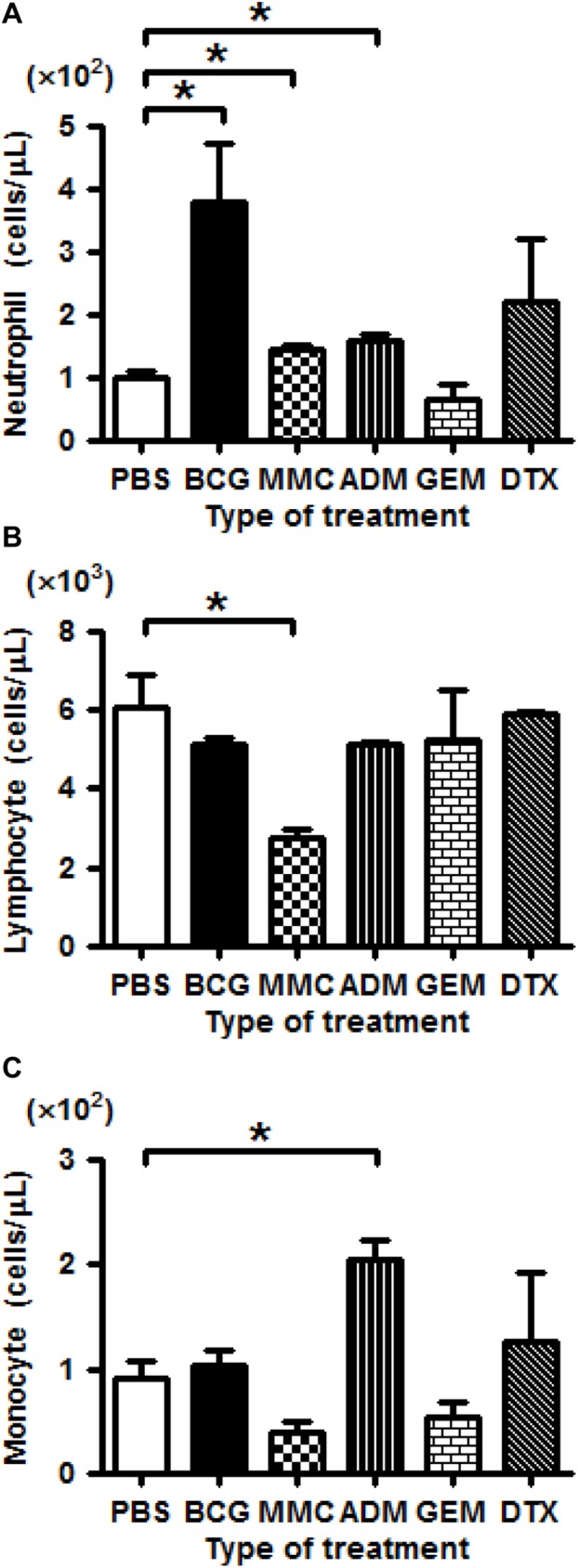
Changes in the populations of blood cells caused by intravesical treatment. (A) The number of neutrophils is increased in BCG, MMC, and ADM. (B) The number of lymphocytes is decreased in MMC. (C) The number of monocytes is increased in ADM (Mann-Whitney U test; * = *P*<0.05).

A higher population of neutrophils was observed in mice treated with BCG, MMC, and ADM compared with the control group (*P*<0.01, *P*<0.01, *P*<0.01, respectively; Fig 6A, S8 Table). The population of lymphocytes was similar to that of the control group, but was significantly lower upon MMC treatment (*P*<0.01; Fig 6B, S8 Table). Finally, the number of monocytes rose significantly upon intravesical treatment with ADM compared with the control (*P* = 0.013; Fig 6C, S8 Table).

## Discussion

**Fig 7:**
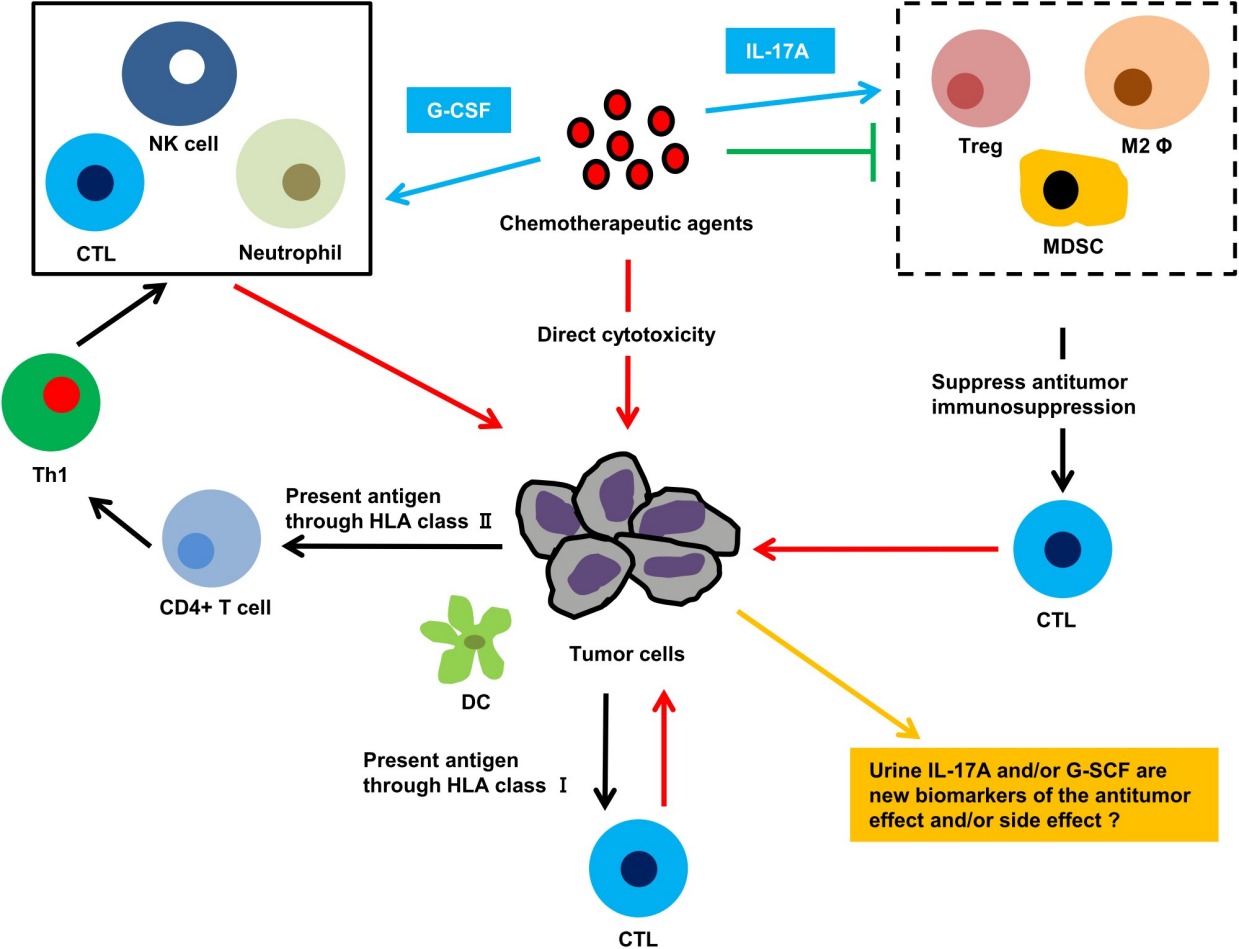
Schematic summary of the study. Intravesical treatment with chemotherapeutic agents shows anti-tumor activity through direct cytotoxicity and indirectly through modulation of anti-tumor immunity. Cytotoxic T cells recognize a cancer-specific antigen from apoptotic cancer cells and eliminate these cells. In addition, dendritic cells (DC) capture the antigen and present it to CD4+ T cells. Activated CD4+ T cells produce cytokines resulting in activation of helper T1 (Th1) cells and recruitment of cytotoxic T cells, NK cells, and macrophages through cytokines. IL-17A and G-CSF suppress protumoral immunity and enhance anti-tumor immunity (red allow: anti-tumor effect, black allow: general immunoresponse, blue allow: regulation by serum IL-17A and G-CSF, green allow: inhibition of immune-related cells, yellow allow: urine IL-17A and G-CSF as markers).

Our study shows that all tested chemotherapeutic agents had an anti-tumor effect, similar to that of BCG, against an orthotopic bladder cancer model. Moreover, some agents, such as MMC and ADM, elicited unique changes to immune cells, including a reduction of NK cells, M2 macrophages, and Tregs. In human samples M2 macrophage and Tregs were reduced by the treatment of MMC and ADM even if it recurred, suggesting the effect of these drugs may be memorized and last for a while. Although intravesical treatment was originally a local therapy against NMIBC, an ELISArray assay of serum revealed a systemic immunoreaction characterized by induction of inflammatory cytokines, such as IL-1A, IL-4, IL-10, IL-17A, and G-CSF. Thus, we hypothesize that intravesical chemotherapeutic agents bind to tumor cells, eliciting a direct anti-tumor effect. Simultaneously, tumor cells that have incorporated the chemotherapeutic agents present a cancer-specific antigen to antigen-presenting cells resulting in an indirect anti-tumor effect (Fig 7).

The main anti-tumor action of chemotherapeutic drugs comes from blocking DNA synthesis (acetylation or inhibition of topoisomerase), RNA synthesis (inhibition of RNA polymerase), and cell division (inhibition of microtubule depolymerization). The present study shows that chemotherapeutic agents delivered by intravesical treatment are capable of systemically and locally altering the levels of immune cells and inflammatory cytokines, resulting in indirect anti-tumor activity. Cytotoxic T lymphocytes (CTL) play an important role in tumor immunity, stimulated by Th1 cells, IL-2, and IFN-γ [20]. Tumors, in turn, inhibit this anti-tumor activity by producing immunosuppressive cytokines, such as IL-10 and TNF-β, and inducing immunosuppressive cells, such as Tregs, tumor-associated macrophages (TAMs), and myeloid-derived suppressor cells (MDSC) [21–24]. Tregs are mostly involved in controlling cancer immune escape and their presence can be used for cancer prognosis [25–27]. The present study shows a significant reduction in Treg expression in the bladder upon MMC and ADM administration. This suggests that MMC and ADM reverse the immune suppression caused by cancer cells. A combined treatment with BCG and MMC inhibits the growth of bladder cancer more effectively than each individual treatment alone [28]. Svatek *et al*. reported that sequential intravesical treatment of NMIBC with BCG and MMC increased the concentration of urinary IL-8 compared with BCG monotherapy and directed TAMs towards the production of antitumoral M1 macrophages [29]. ADM has also been shown to indirectly promote an anti-tumor immunoreaction [30–34], through the proliferation of tumor-specific CD8+ T cells [32] or the increased permeability of tumor cells to CTL-produced granzyme B [33]. Moreover, Alizadeh *et al*. showed that ADM selectively removed MDSCs, impaired the suppressive function of residual MDSCs, and enhanced the rate of NK and CTL expression of perforin, granzyme B, and IFN-γ by NK cells and CTL [34]. Whereas intravesical treatment with MMC or ADM promotes a change in anti-tumor immunity, GEM and DTX do not appear to have any effect. This observation contrasts with previous reports on GEM selectively depleting Tregs in cancer, improving anti-tumor immunoreaction [35–37], or suppressing Treg induction in peripheral blood mononuclear cells. Similarly, Turk *et al*. [38] reported that DTX increased the number of CD4+ and CD8+ T cells, whereas Li *et al*. reported that treatment with DTX reduced the number of Tregs, increased the secretion of INF-γ and lowered that of TGF-β in non-small cell lung carcinoma [39]. Given that IHC staining of spleens demonstrated that some chemotherapeutic agents induced CT4+ T and NK cells, it is possible that intravesical treatment with GEM or DTX may nevertheless result in anti-tumor activity. Further studies are needed to reveal the association between intravesical chemotherapies and immune-related cells.

IL-17A is a T helper 17 (Th17)-derived pro-inflammatory cytokine that induces mobilization of neutrophils [40]. The role of IL-17A in cancer remains controversial. On the one hand, IL-17A has been shown to induce vascular endothelial growth factor, suppress apoptosis of various tumor cell lines *in vitro*, and directly promote tumor growth [41–44]. On the other hand, Kryczek *et al*. [45] reported that tumor growth and lung metastasis were enhanced in IL-17A-deficient mice, whereas Hirahara *et al*. [46] reported that IL-17A augmented the expression of the major histocompatibility complex class I and class II antigens and induced anti-tumor immunity. In the present study, serum IL-17A was high in the BCG and MMC groups, both of which displayed anti-tumor activity. Consequently, we suggest that IL-17A induced by intravesical chemotherapy plays a role in enhancing anti-tumor immunity towards NMIBC. We hypothesize that IL-17A promotes excretion of G-CSF by stromal cells and the subsequent induction of neutrophils leads to anti-tumor activity through Fas and/or TRAIL apoptotic pathways. In addition, IL-17A may be involved in immunomodulation by suppressing immune-related cells, which play a role in inhibiting anti-tumor immunity. Given that serum G-CSF increased in the BCG, MMC, ADM, and DTX groups, all of which presented anti-tumor activity in a BBN-induced bladder cancer model, we suggest that the anti-tumor activity of G-CSF relies on neutrophil induction.

Saban *et al*. first reported that the therapeutic benefit of intravesical treatment with BCG involved induction of urinary IL-17A and Th17. Thus, the balance of Th1, Th2, and Th17 is important for anti-tumor activity [47]. Shintani *et al*. reported that the concentration of urinary G-CSF was higher in the non-recurrent group treated with intravesical BCG than in the recurrent group [48]. Although further experiments are needed, IL-17A and G-CSF may represent novel biomarkers and a new therapeutic target for the intravesical treatment of NMIBC.

The unique populations of neutrophils, lymphocytes, and monocytes in peripheral blood were also assessed in this study. Recently, neutrophil:lymphocyte and lymphocyte:monocyte ratios have been reported as predictive factors for bladder cancer [49, 50]. Here, chemotherapeutic agents induced or reduced the number of blood cells compared to the control. These findings may help us evaluate the effectiveness and/or side effects of intravesical treatment and lead to better management of NMIBC. Also, the population of blood cells was induced by cytokines, such as IL-17A and G-CSF. Further experiments are needed to explain these changes and to develop a novel combined or sequential therapy with BCG and chemotherapeutic agents.

This study has some limitations. First, the BBN intake varied among the mice, which could cause variability in the tumor size and/or tumor behavior for each mouse. Second, a single dose was tested for each treatment, so we did not evaluate the dose-dependent effect in this study. Third, with regard to IHC analysis, we conducted blinded assessment by multiple investigators. It might be more objective and reliable to use an automated quantitative tool. Last, the sample size of human bladder tissues were small as the number of patients treated with MMC or ADM was limited in our institution. The careful interpretation needs to be done for the confirmation study using human samples.

## Conclusion

Intravesical treatment with chemotherapeutic agents demonstrates that their anti-tumor activity is similar to that achieved by BCG in a BBN-induced bladder cancer mouse model. Intravesical chemotherapy suppresses protumoral immunity, enhances anti-tumor immunity, systemically and locally affects the induction of cytokines and chemokines, and alters the population of neutrophils, lymphocytes, and monocytes. Improved knowledge about the immune response induced by chemotherapeutic agents can lead to better outcomes and fewer side effects in NMIBC intravesical treatment. Further investigation, including clinical studies, will be required to develop a personalized strategy for intravesical chemotherapy based on the microenvironment status of the bladder.

## Supporting information

S1 Fig Relative expression level of each immunological marker (CD4, CD8, CD56, CD204, Foxp3, PD-L1) in treatment groups and PBS control. CD4+ and CD8+ T cells are induced by BCG. NK cells are induced by BCG and reduced by MMC. M2 macrophages and regulatory T cells are reduced by MMC and ADM (Mann-Whitney U test; * = *P*<0.05).
(TIF)

S2 Fig Representative images of spleen samples from each treatment group stained with six immunological markers (CD4, CD8, CD56, CD204, Foxp3, PD-L1). (TIF)

S3 Fig Relative expression level of each immunological marker (CD4, CD8, CD56, CD204, Foxp3, PD-L1) in treatment groups and PBS control. CD4+ T cells are induced by BCG, MMC, and DTX. NK cells are induced by BCG, MMC, ADM, and GEM (Mann-Whitney U test; * = *P*<0.05).
(TIF)

S1 Table Patients`background. Total twelve patients are investigated (4 patients in each group).
(DOCX)

S2 Table Raw data of resected bladder weight. Raw data of Fig 1C.
(XLSX)

S3 Table Raw data of the number of positive cells stained with each marker using mouse bladder samples. Raw data of S1 Fig.
(XLSX)

S4 Table Raw data of the number of positive cells stained with each marker using mouse spleen samples. Raw data of S3 Fig.
(XLSX)

S5 Table Raw data of IHC analysis using human bladder samples. Raw data of Fig 3.
(XLSX)

S6 Table Transition of IL-17A and G-CSF in urine. IL-17A and G-CSF are increased or decreased in intravesical chemotherapy.
(DOCX)

S7 Table Raw data of urine concentration of IL-A and G-CSF. Raw data of Fig 5 and S6 Table.
(XLSX)

S8 Table Raw data of the number of blood cells (neutrophils, lymphocytes, and monocytes). Raw data of Fig 6.
(XLSX)

## Abbreviations

UCB: urothelial carcinoma of the bladder
NMIBC: non-muscle invasive bladder cancer
TURBT: transurethral resection of the bladder tumor
BCG: Bacillus Calmette-Guerin
MMC: mitomycin C
GEM: gemcitabine
DTX: docetaxel
MIBC: muscle invasive bladder cancer
IL: interleukin
TNF: tumor necrosis factor
GM-CSF: granulocyte macrophage colony stimulating factor
Th1: T-helper 1
Th2: T-helper 2
TRAIL: tumor necrosis factor-related apoptosis-inducing ligand
Fas: fatty-acid synthetase
BBN: N-butyl-N-(4-hydroxybutyl) nitrosamine
ADM: adriamycin
PBS: phosphate buffered saline
CD: Cluster designation
Foxp3: Forkhead box p3
PD-L1: Programmed cell death-ligand 1
HPF: high power field
IFN: interferon
G-CSF: granulocyte colony stimulating factor
Treg: regulatory T cells
CTL: cytotoxic T lymphocytes
TAM: tumor-associated macrophage
MDSC: myeloid-derived suppressor cell
Th17: T-helper 17

## References

[1] Siegel R, Ma J, Zou Z, Jemal A. Cancer statics, 2014. CA Cancer J. Clin. 2014; 64: 9–29. doi: 10.3322/caac.21208 24399786 10.3322/caac.21208

[2] Miyake M, Fujimoto K, Anai S, Ohnishi S, Nakai Y, Inoue T, et al Clinical significance of heme oxygenase-1 expression in non-muscle-invasive bladder cancer. Urol. Int 2010;85:355–363. doi: 10.1159/000317785 20733275 10.1159/000317785

[3] Nepple KG, O’Donnell MA. The optimal management of T1 high-grade bladder cancer. Can Urol Assoc J 2009;3:S188–192. 20019983 PMC2792452

[4] Hendricksen K, Witjes JA. Current strategies for first and second line intravesical therapy for nonmascle invasive bladder cancer. Curr Opin Urol 2007;17:352–357 doi: 10.1097/MOU.0b013e3281c55f2b 17762630 10.1097/MOU.0b013e3281c55f2b

[5] Barlow LJ, McKiernan JM, Benson MC. Long-term survival outcomes with intravesical docetaxel for recurrent nonmuscle invasive bladder cancer after previous bacillus Calmette-Guérin therapy. J Urol 2013;189:834–839 doi: 10.1016/j.juro.2012.10.068 23123371 10.1016/j.juro.2012.10.068

[6] Babjuk M, Burger M, Zigeuner R, Shariat SF, van Rhijn BW, Comperat E, et al EAU guidelines on non-muscle-invasive urothelial carcinoma of the bladder: update 2013. Eur Urol 2013;64:639–653 doi: 10.1016/j.eururo.2013.06.003 23827737 10.1016/j.eururo.2013.06.003

[7] Clark PE, Agarwal N, Biagioli MC, Eisenberger MA, Greenberg RE, Herr HW, et al National Comprehensive Cancer Network (NCCN). Bladder cancer. J Natl Compr Canc Netw 2013;11:446–475 23584347 10.6004/jnccn.2013.0059

[8] Peyromaure M, Guerin F, Amsellem-Ouazana D, Saighi D, Debre B, Zerbib M. Intravesical bacillus Calmette-Guerin therapy for stage T1 grade 3 transitional cell carcinoma of the bladder: recurrence, progression and survival in a study of 57 patients. J Urol 2003;169:2110–2112. doi: 10.1097/01.ju.0000066840.42991.4a 12771729 10.1097/01.ju.0000066840.42991.4a

[9] Shahin O, Thalmann GN, Rentsch C, Mazzucchelli L, Studer UE. A retrospective analysis of 153 patients treated with or without intravesical bacillus Calmette-Guerin for primary stage T1 grade 3 bladder cancer: recurrence, progression and survival. J Urol 2003;169:96–100. doi: 10.1097/01.ju.0000035543.69161.58 12478112 10.1016/S0022-5347(05)64044-X

[10] Herr HW. Intravesical bacillus Calmette-Guerin outcomes in patients with bladder cancer and asymptomatic bacteriuria. J Urol 2012;187:435–437 doi: 10.1016/j.juro.2011.10.032 22177154 10.1016/j.juro.2011.10.032

[11] Kikuchi E, Fujimoto H, Mizutani Y, Okajima E, Koga H, Hinotsu S, et al Cancer Registration Committee of the Japanese Urological Association. Clinical outcome of tumor recurrence for Ta, T1 non-muscle invasive bladder cancer from the data on registered bladder cancer patients in Japan: 1999–2001 report from the Japanese Urological Association. Int J Urol 2009;16:279–286. doi: 10.1111/j.1442-2042.2008.02235.x 19207609 10.1111/j.1442-2042.2008.02235.x

[12] Herr HW, Morales A. History of Bacillus Calmette-Guerin and bladder cancer: an immunotherapy success story. J Urol 2008;179:53–56 doi: 10.1016/j.juro.2007.08.122 17997439 10.1016/j.juro.2007.08.122

[13] Becich MJ, Carroll S, Ratliff TL. Internalization of bacilli Calmette-Guerin by bladder tumor cells. J Urol 1991; 145(6):1316–1324 2033723 10.1016/s0022-5347(17)38622-6

[14] Bevers RF, Kurth KH, Schamhart DH. Role of urothelial cells in BCG immunotherapy for superficial bladder cancer. Br J Cancer 2004;91:607–612 doi: 10.1038/sj.bjc.6602026 15266312 10.1038/sj.bjc.6602026 PMC2364784

[15] Saint F, Kurth N, Maille P, Vordos D, Hoznek A, Soyeux P, et al Urinary IL-2 assay for monitoring intravesical bacillus Calmette-Guerin response of superficial bladder cancer during induction course and maintenance therapy. Int J Cancer 2003;107:434–440 doi: 10.1002/ijc.11352 14506744 10.1002/ijc.11352

[16] Ludwig AT, Moore JM, Luo Y, Chen X, Saltsgaver NA, O’Donnell MA, et al Tumor necrosis factor-related apoptosis-inducing ligand: a novel mechanism for Bacillus Calmette-Guerin-induced anti-tumor activity. Cancer Res 2004;64(10):3386–3390 doi: 10.1158/0008-5472.CAN-04-0374 15150089 10.1158/0008-5472.CAN-04-0374

[17] Simmons MP, Nauseef WM, Griffith TS. Neutrophils and TRAIL: insights into BCG immunotherapy for bladder cancer. Immunologic Res 2007;39:79–93 10.1007/s12026-007-0084-1 17917057

[18] Bohle A, Gerdes J, Ulmer AJ, Hofstetter AG, Flad HD. Effects of local bacillus Calmette-Guerin therapy in patients with bladder carcinoma on immunocompetent cells of the bladder wall. J Urol 1990;144:53–58 2359181 10.1016/s0022-5347(17)39365-5

[19] Askeland EJ, Newton MR, O’Donnell MA, Luo Y. Bladder Cancer Immunotherapy: BCG and Beyond. Adv Urol 2012;181987. 10.1155/2012/181987 PMC3388311 22778725

[20] Kitamura H, Tsukamoto T. Immunotherapy for urothelial carcinoma: current status and perspectives. Cancers (Basel). 2011;3:3055–3072 24212945 10.3390/cancers3033055 PMC3759186

[21] Loskog A, Ninalga C, Paul-Wetterberg G, de la Torre M, Malmstrom PU, Totterman TH. Human bladder carcinoma is dominated by T-regulatory cells and Th1 inhibitory cytokines. J Urol 2007;177:353–358 doi: 10.1016/j.juro.2006.08.078 17162090 10.1016/j.juro.2006.08.078

[22] Sakaguchi S, Sakaguchi N, Shimizu J, Yamazaki S, Sakihama T, Itoh M, et al Immunologic tolerance maintained by CD25+ CD4+ regulatory T cells: their common role in controlling autoimmunity, tumor immunity, and transplantation tolerance. Immunol Rev 2001;182:18–32 11722621 10.1034/j.1600-065x.2001.1820102.x

[23] Gabrilovich DI, Nagarai S. Myeloid-derived suppressor cells as regulators of the immune system. Nat Rev Immunol 2009;9:162–174 doi: 10.1038/nri2506 19197294 10.1038/nri2506 PMC2828349

[24] Zhao Y, Wang D, Xu T, Liu P, Cao Y, Wang Y, et al Bladder cancer cells re-educate TAMs through lactate shuttling in the microfluidic cancer microenvironment. Oncotarget 2015;6:39196–39210 doi: 10.18632/oncotarget.5538 26474279 10.18632/oncotarget.5538 PMC4770766

[25] Terabe M, Berzofsky JA. Immunoregulatory T cells in tumor immunity. Vurr Opin Immunol 2004;16:157–162 10.1016/j.coi.2004.01.010 15023407

[26] Beyer M, Schultze JL. Regulatory T cells in cancer. Blood 2006;108(3):804–811 doi: 10.1182/blood-2006-02-002774 16861339 10.1182/blood-2006-02-002774

[27] Nakamura R, Sakakibara M, Nagashima T, Sangai T, Arai M, Fujimori T, et al Accumulation of regulatory T cells in sentinel lymph nodes is a prognostic predictor in patients with node-negative breast cancer. Eur J Cancer 2009;45:2123–2131 doi: 10.1016/j.ejca.2009.03.024 19398324 10.1016/j.ejca.2009.03.024

[28] Matsushima M, Horinaga M, Fukuyama R, Yanaihara H, Kikuchi E, Kawachi M, et al Enhanced antitumor effect of combination intravesical mitomycin C and bacillus Calmette-Guerin therapy in an orthotopic bladder cancer model. Oncol Lett 2011;2:13–19 doi: 10.3892/ol.2010.217 22870122 10.3892/ol.2010.217 PMC3412510

[29] Svatek RS, Zhao XR, Morales EE, Jha MK, Tseng TY, Hugen CM, et al Sequential intravesical mitomycin plus Bacillus Calmette-Guérin for non-muscle-invasive urothelial bladder carcinoma: translational and phase I clinical trial. Clin Cancer Res 2015;21;303–311 doi: 10.1158/1078-0432.CCR-14-1781 25424854 10.1158/1078-0432.CCR-14-1781 PMC4297588

[30] Mattarollo SR, Loi S, Duret H, Ma Y, Zitvogel L, Smyth MJ. Pivotal role of innate and adaptive immunity in anthracycline chemotherapy of established tumors. Cancer Res 2011;71:4809–4820 doi: 10.1158/0008-5472.CAN-11-0753 21646474 10.1158/0008-5472.CAN-11-0753

[31] Bandyopadhyay A1, Wang L, Agyin J, Tang Y, Lin S, Yeh IT, et al Doxorubicin in combination with a small TGFbeta inhibitor: a potential novel therapy for metastatic breast cancer in mouse models. PloS one 2010;5: e10365 doi: 10.1371/journal.pone.0010365 20442777 10.1371/journal.pone.0010365 PMC2860989

[32] Obeid M, Tesniere A, Ghiringhelli F, Fimia GM, Apetoh L, Perfettini JL, et al Calreticulin exposure dictates the immunogenicity of cancer cell death. Nat Med 2007;13:54–61 doi: 10.1038/nm1523 17187072 10.1038/nm1523

[33] Ramakrishnan R, Assudani D, Nagaraj S, Hunter T, Cho HI, Antonia S, et al Chemotherapy enhances tumor cell susceptibility to CTL-medeated killing during cancer immunotherapy in mice. J Clin Invest 2010;120:1111–1124 doi: 10.1172/JCI40269 20234093 10.1172/JCI40269 PMC2846048

[34] Alizadeh D, Trad M, Hanke NT, Larmonier CB, Janikashvili N, Bonnnotte B, et al Doxorubicin eliminates myeloid-derived suppressor cells and enhances the efficacy of adoptive T-cell transfer in breast cancer. Cancer Res 2014;74:104–118 doi: 10.1158/0008-5472.CAN-13-1545 24197130 10.1158/0008-5472.CAN-13-1545 PMC3896092

[35] Shevchenko I, Karakhanova S, Soltek S, Link J, Bayry J, Werner J, et al Low-dose gemcitabine depletes regulatory T cells and improves survival in the orthotopic Panc02 model of pancreatic cancer. Int J Cancer 2013;133:98–107 doi: 10.1002/ijc.27990 23233419 10.1002/ijc.27990

[36] Zheng Y, Dou Y, Duan L, Cong C, Gao A, Lai Q, et al Using chemo-drugs or irradiation to break immune tolerance and facilitate immunotherapy in solid cancer. Cell Immunol 2015;294:54–59 doi: 10.1016/j.cellimm.2015.02.003 25687508 10.1016/j.cellimm.2015.02.003

[37] Kan S, Hazama S, Maeda K, Inoue Y, Homma S, Koido S, et al Suppressive effects of cyclophosphamide and gemcitabine on regulatory T-cell induction in vitro. Anticancer Res 2012;32:5363–5369 23225438

[38] Turk MJ, Guevara-Patino JA, Rizzuto GA, Engelhorn ME, Sakaguchi S, Houghton AN. Concomitant tumor immunity to a poorly immunogenic melanoma is prevented by regulatory T cells. J Exp Med 2004;200(6):771–782 doi: 10.1084/jem.20041130 15381730 10.1084/jem.20041130 PMC2211964

[39] Li JY, Duan XF, Wang LP, Xu YJ, Huang L, Zhang TF, et al Selective depletion of regulatory T cell subsets by docetaxel treatment in patients with nonsmall cell lung cancer. J Immunol Res 2014;2014:286170 doi: 10.1155/2014/286170 24868562 10.1155/2014/286170 PMC4020463

[40] Kolls JK, Linden A. Interleukin-17 family members and inflammation. Immunity 2004;21:467–476 doi: 10.1016/j.immuni.2004.08.018 15485625 10.1016/j.immuni.2004.08.018

[41] Takahashi H, Numasaki M, Lotze MT, Sasaki H. Interleukin-17 enhances bFGF-, HGF- and VEGF-induced growth of vascular endothelial cells. Immunol Lett 2005;98:189–193 doi: 10.1016/j.imlet.2004.11.012 15860217 10.1016/j.imlet.2004.11.012

[42] Hayata K, Iwahashi M, Ojima T, Katsuda M, Iida T, Nakamori M, et al Inhibition of IL-17A in tumor microenvironment augments cytotoxicity of tumor-infiltrating lymphocytes in tumor-bearing mice. Plos One 2013;8:e53131 doi: 10.1371/journal.pone.0053131 23372655 10.1371/journal.pone.0053131 PMC3556079

[43] Numasaki M, Watanabe M, Suzuki T, Takahashi H, Nakamura A, McAllister F, et al IL-17 enhances the net angiogenic activity and in vivo growth of human non-small cell lung cancer in SCID mice through promoting CXCR-2-dependent angiogenesis. J Immunol 2005;175:6177–6189 16237115 10.4049/jimmunol.175.9.6177

[44] Nam JS, Terabe M, Kang MJ, Chae H, Voong N, Yang YA, et al Transforming growth factor beta subverts the immune system into directly promoting tumor growth through interleukin-17. Cancer Res 2008;68:3915–3923 doi: 10.1158/0008-5472.CAN-08-0206 18483277 10.1158/0008-5472.CAN-08-0206 PMC2586596

[45] Kryczek I, Wei S, Szeliga W, Vatan L, Zou W. Endogenous IL-17 contributes to reduced tumor growth and metastasis. Blood 2009;114:357–359 doi: 10.1182/blood-2008-09-177360 19289853 10.1182/blood-2008-09-177360 PMC2714210

[46] Hirahara N, Nio Y, Sasaki S, Minari Y, Takamura M, Iguchi C, et al Inoculation of human interleukin-17 gene-transfected Meth-A fibrosarcoma cells induces T cell-dependent tumor-specific immunity in mice. Oncology 2001;61:79–89 doi: 55357 11474253 10.1159/000055357

[47] Saban MR, Simpson C, Davis C, Wallis G, Knowlton N, Frank MB, et al Discriminators of mouse bladder response to intravesical Bacillus Calmette-Guerin (BCG). BMC Immunol 2007;8:6 doi: 10.1186/1471-2172-8-6 17506885 10.1186/1471-2172-8-6 PMC1891101

[48] Shintani Y, Sawada Y, Inagaki T, Kohjimoto Y, Ueoka Y, Shinka T. Intravesical instillation therapy with bacillus Calmette-Guérin for superficial bladder cancer: study of the mechanism of bacillus Calmette-Guérin immunotherapy. Int J Urol 2007;14:140–146 doi: 10.1111/j.1442-2042.2007.01696.x 17302571 10.1111/j.1442-2042.2007.01696.x

[49] Temraz S, Mukherji D, Farhat ZA, Nasr R, Charafeddine M, Shahait M, et al Preoperative lymphocyte-to-monocyte ratio predicts clinical outcome in patients undergoing radical cystectomy for transitional cell carcinoma of the bladder: a retrospective analysis. BMC Urol 2014;14:76 doi: 10.1186/1471-2490-14-76 25234356 10.1186/1471-2490-14-76 PMC4171398

[50] Wei Y, Jiang YZ, Qian WH. Prognostic role of NLR in urinary cancers: a meta-analysis. PLoS One 2014;9:e92079 doi: 10.1371/journal.pone.0092079 24642859 10.1371/journal.pone.0092079 PMC3958449

