# Supplementary figures and images for: Topical and systemic immunoreaction triggered by intravesical chemotherapy in an N-butyl-N-(4-hydroxybutyl) nitorosamine induced bladder cancer mouse model
Source: PLoS One. 2017 Apr 13;12(4):e0175494. doi: 10.1371/journal.pone.0175494 (PMC5391151; doi:10.1371/journal.pone.0175494)

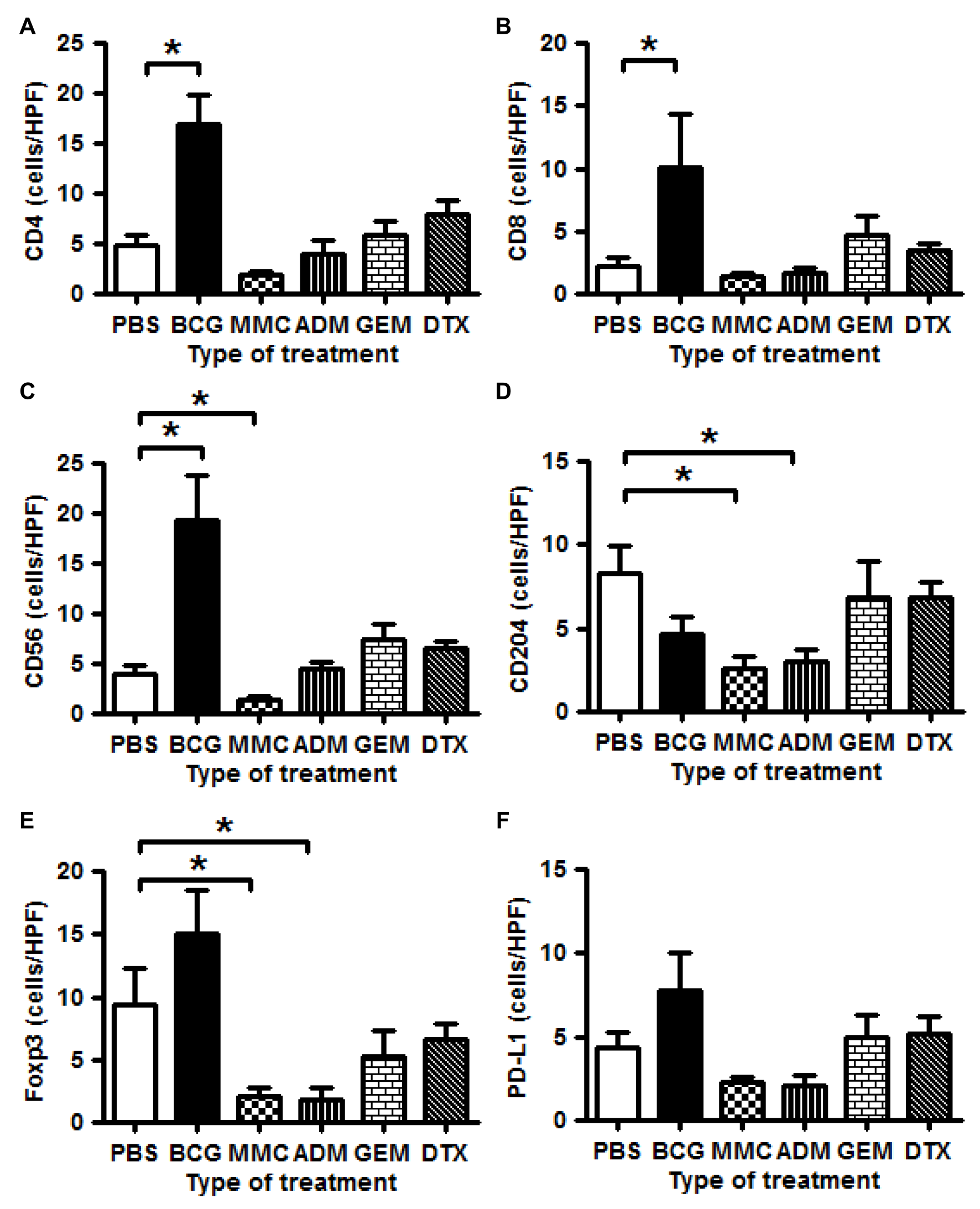

Supplement: S1 Fig — CD4+ and CD8+ T cells are induced by BCG. NK cells are induced by BCG and reduced by MMC. M2 macrophages and regulatory T cells are reduced by MMC and ADM (Mann-Whitney U test; * = P<0.05). (TIF) [file pone.0175494.s001.tif]

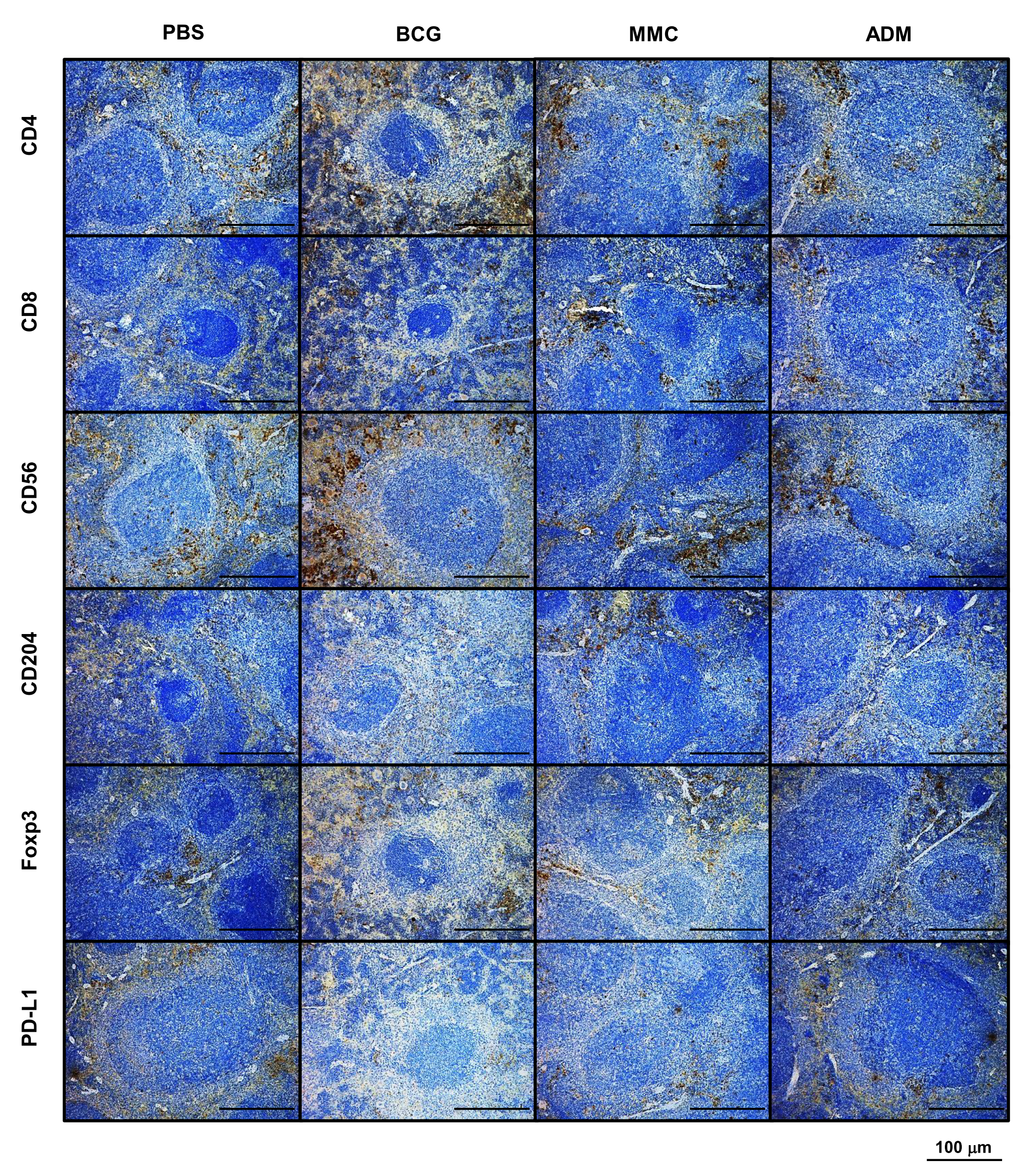

Supplement: S2 Fig — (TIF) [file pone.0175494.s002.tif]

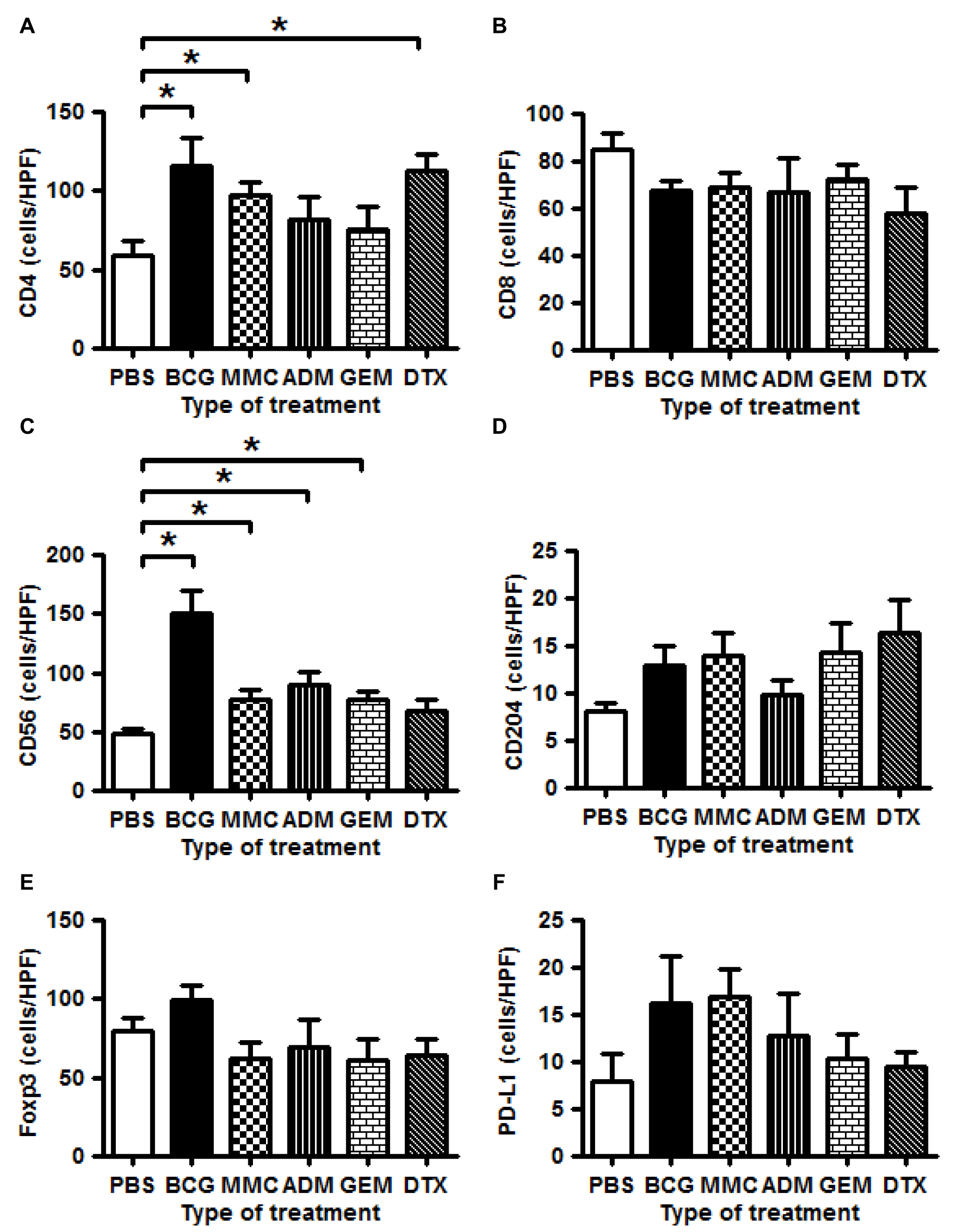

Supplement: S3 Fig — CD4+ T cells are induced by BCG, MMC, and DTX. NK cells are induced by BCG, MMC, ADM, and GEM (Mann-Whitney U test; * = P<0.05). (TIF) [file pone.0175494.s003.tif]
