# Supplementary material for: Topical and systemic immunoreaction triggered by intravesical chemotherapy in an N-butyl-N-(4-hydroxybutyl) nitorosamine induced bladder cancer mouse model
Source: PLoS One. 2017 Apr 13;12(4):e0175494. doi: 10.1371/journal.pone.0175494 (PMC5391151; doi:10.1371/journal.pone.0175494)
Supplement: S1 Table — Total twelve patients are investigated (4 patients in each group). (DOCX) [file pone.0175494.s004.docx]

| **Table S1. Patients` background** | | | | | | |
| --- | --- | --- | --- | --- | --- | --- |
| **Variables** | | **Number of patients** | **Type of treatment** | | | ***P* value** |
|  |  |  | **BCG** | **MMC** | **ADM** |  |
| **Total** |  | **12** | **4** | **4** | **4** |  |
| **Gender** |  |  |  |  |  | **0.69 †** |
|  | **Male** | **9** | **2** | **3** | **3** |  |
|  | **Female** | **3** | **2** | **1** | **1** |  |
| **Age (at initial TURBT)** | |  |  |  |  | **0.45 ‡** |
| **Median (IQR)** | | **71 (62-84)** | **72 (64-77)** | **69 (62-76)** | **73 (68-84)** |  |
| **TURBT = transurethral resection of bladder tumor; IQR = interquartile range; BCG = bacillus Calmette-Guerin; MMC = mitomycin C; ADM = adriamycin; † Chi-square test; ‡ Mann-Whitney U test.** | | | | | | |
|  |  |  |  |  |  |  |
|  |  |  |  |  |  |  |
