# Supplementary material for: Topical and systemic immunoreaction triggered by intravesical chemotherapy in an N-butyl-N-(4-hydroxybutyl) nitorosamine induced bladder cancer mouse model
Source: PLoS One. 2017 Apr 13;12(4):e0175494. doi: 10.1371/journal.pone.0175494 (PMC5391151; doi:10.1371/journal.pone.0175494)
Supplement: S6 Table — IL-17A and G-CSF are increased or decreased in intravesical chemotherapy. (DOCX) [file pone.0175494.s009.docx]

| **S2 Table. Transition of IL-17A and G-CSF in urine** | | | | | | | |
| --- | --- | --- | --- | --- | --- | --- | --- |
|  | |  | **Time of urine sampling during treatment** | | | | |
|  | |  | **Before Tx** | **1W** | **2W** | **3W** | **4W** |
| **IL-17A pg/mL (mean, SD)** | | |  |  |  |  |  |
|  | **PBS** | | **7.8 ± 1.2** | **18.4 ± 2.9** | **13.9 ± 1.7** | **15.8 ± 2.1** | **18.2 ± 2.1** |
|  | **BCG** | | **7.8 ± 2.0** | **22.6 ± 2.5** | **24.9 ± 4.1 *** | **29.1 ± 3.7 *** | **25.1 ± 4.9** |
|  | **MMC** | | **9.6 ± 1.1** | **19.7 ± 5.0** | **9.6 ± 1.8** | **9.0 ± 1.6 *** | **11.1 ± 2.5 *** |
|  | **ADM** | | **7.5 ± 2.1** | **20.4 ± 3.7** | **15.8 ± 1.7** | **14.4 ± 4.1** | **17.3 ± 4.2** |
|  | **GEM** | | **8.5 ± 2.9** | **29.6 ± 4.2 *** | **24.2 ± 4.5 *** | **17.7 ± 3.3** | **16.4 ± 3.3** |
|  | **DTX** | | **9.0 ± 4.3** | **22.3 ± 1.9** | **21.8 ± 2.5 *** | **21.4 ± 4.2** | **23.2 ± 2.1** |
| **G-CSF pg/mL (mean, SD)** | | |  |  |  |  |  |
|  | **PBS** | | **1.9 ± 0.3** | **4.0 ± 0.8** | **0.9 ± 0.1** | **5.6 ± 0.8** | **13.3 ± 1.7** |
|  | **BCG** | | **2.7 ± 0.5** | **15.9 ± 1.6 *** | **3.6 ± 0.5 *** | **4.9 ± 1.8** | **18.3 ± 1.7 *** |
|  | **MMC** | | **3.3 ± 0.8** | **35.3 ± 2.5 *** | **83.0 ± 2.9 *** | **19.4 ± 2.9 *** | **10.2 ± 0.9** |
|  | **ADM** | | **2.1 ± 0.6** | **7.3 ± 2.1** | **3.2 ± 1.3** | **11.7 ± 1.7 *** | **11.3 ± 1.7** |
|  | **GEM** | | **2.0 ± 0.8** | **11.5 ± 1.2 *** | **9.0 ± 0.8 *** | **3.2 ± 0.6 *** | **2.7 ± 0.9 *** |
|  | **DTX** | | **2.8 ± 0.9** | **14.8 ± 1.7 *** | **17.4 ± 2.0 *** | **29.2 ± 4.9 *** | **9.7 ± 1.7** |
| **ADM = adriamycin; BCG = bacillus Calmette-Guerin; DTX = docetaxel; G-CSF = granulocyte colony stimulating factor; GEM = gemcitabine; IL = interleukin; MMC = mitomycin C; PBS = phosphate buffered saline; Tx = treatment; W = week after the initial treatment; * = significant difference compared to PBS control at the same time point (< 0.05); 15 samples were analyzed at one time point in each group Student’s t-test** | | | | | | | |
|  |  |  |  |  |  |  |  |
|  |  |  |  |  |  |  |  |
|  |  |  |  |  |  |  |  |
